# Co-Development of Diagnostic Vectors to Support Targeted Therapies and Theranostics: Essential Tools in Personalized Cancer Therapy

**DOI:** 10.3389/fonc.2014.00141

**Published:** 2014-06-13

**Authors:** Nicholas C. Nicolaides, Daniel J. O’Shannessy, Earl Albone, Luigi Grasso

**Affiliations:** ^1^Department of Translational Medicine and Diagnostics, Morphotek Inc., Exton, PA, USA

**Keywords:** companion diagnostics, CDx, co-development, TM601, theranostics, nanoparticles, naturally occurring proteins, personalized medicine

## Abstract

Novel technologies are being developed to improve patient therapy through the identification of targets and surrogate molecular signatures that can help direct appropriate treatment regimens for efficacy and drug safety. This is particularly the case in oncology whereby patient tumor and biofluids are routinely isolated and analyzed for genetic, immunohistochemical, and/or soluble markers to determine if a predictive biomarker signature (i.e., mutated gene product, differentially expressed protein, altered cell surface antigen, etc.) exists as a means for selecting optimal treatment. These biomarkers may be drug-specific targets and/or differentially expressed nucleic acids, proteins, or cell lineage profiles that can directly affect the patient’s disease tissue or immune response to a therapeutic regimen. Improvements in diagnostics that can prescreen predictive response biomarker profiles will continue to optimize the ability to enhance patient therapy via molecularly defined disease-specific treatment. Conversely, patients lacking predictive response biomarkers will no longer needlessly be exposed to drugs that are unlikely to provide clinical benefit, thereby enabling patients to pursue other therapeutic options and lowering overall healthcare costs by avoiding futile treatment. While patient molecular profiling offers a powerful tool to direct treatment options, the difficulty in identifying disease-specific targets or predictive biomarker signatures that stratify a significant fraction within a disease indication remains challenging. A goal for drug developers is to identify and implement new strategies that can rapidly enable the development of beneficial disease-specific therapies for broad patient-specific targeting without the need of tedious predictive biomarker discovery and validation efforts, currently a bottleneck for development timelines. Successful strategies may gain an advantage by employing repurposed, less-expensive existing agents while potentially improving the therapeutic activity of novel, target-specific therapies that may otherwise have off-target toxicities or less efficacy in cells exhibiting certain pathways. Here, we discuss the use of co-developing diagnostic-targeting vectors to identify patients whose malignant tissue can specifically uptake a targeted anti-cancer drug vector prior to treatment. Using this system, a patient can be predetermined in real-time as to whether or not their tumor(s) can specifically uptake a drug-linked diagnostic vector, thus inferring the uptake of a similar vector linked to an anti-cancer agent. If tumor-specific uptake is observed, then the patient may be suitable for drug-linked vector therapy and have a higher likelihood of clinical benefit while patients with no tumor uptake should consider other therapeutic options. This approach offers complementary opportunities to rapidly develop broad tumor-specific agents for use in personalized medicine.

## Personalized Medicine in the Era of Targeted Therapeutic Strategies

Personalized medicine is generally considered as the precise use of drug(s) that can specifically target a patient’s diseased tissue. This is typically achieved by using a diagnostic biomarker or biomolecular signature that can predict clinical response in patients before they are treated (1). In the broadest sense, an example of personalized medicine can be found in the therapeutic management of type 1 diabetes. Patients with this condition are identified initially by abnormal blood glucose levels, whereby glucose serves as a biomarker. Once confirmed by follow-up testing, the disease can be managed by drugs capable of modulating active insulin levels.

In more complex diseases such as cancer, an array of genetic and altered gene product expression changes may be required to determine or predict a patient’s specific response(s) to therapy. Anti-cancer therapeutic strategies include: (i) binding to a specific molecular target of an altered pathway or a sequence-specific gene product that in turn results in selective killing of malignant but not normal cells; (ii) inducing a host immune response against malignant cells; and (iii) enhancing specific uptake of an agent(s) in target cells for disease suppression. Based on their chemical or biochemical nature, targeted anti-cancer agents can be classified into small chemical entities (SCE) capable of disrupting cellular processes such as enzymatic reactions, tubulin polymerization and DNA replication; nucleic acids that can specifically bind a gene product involved in tumor growth and metastasis; and cellular- and protein-based therapies that can specifically target tumor-associated cell surface proteins or soluble ligands (2). All these agents exert their pharmacologic activity by specifically suppressing growth and survival in malignant vs. normal cells.

For targeted cancer therapies, it is important that the compound can specifically bind to a gene product (e.g., ligands/receptors, transcription factors, or enzymes) or a molecular target within a pathway unique to a tumor cell or cells located within the tumor microenvironment that support tumor growth. These agents may include cell and protein-based vaccines, peptides, recombinant proteins, antibodies, antibody fragments, nucleic acid, and target-specific SCEs (2). The development of novel SCEs targeting tumor-altered gene products involved in driving the underlying cause of transformation is expanding across the industry as a result of recent approvals of compounds in this class (3). These include the anti BCR–ABL fusion protein tyrosine kinase inhibitor imatinib (4) and the more recently approved translocated ALK inhibitor, crizotinib (5), and mutant BRAF inhibitor, vemurafenib (6). While this class has shown robust clinical activity in patients containing the altered gene product, the low target frequency of the latter two compounds has minimized their utility in the greater patient population. Other classes of targeted therapies include antibody and protein-based agents that can specifically bind cell surface proteins on tumor cells and in turn block or activate receptor signaling, induce programed cell death and/or induce immune-mediated cytotoxicity. In all cases, patients may be prescreened to determine if their tumor expresses an agent-specific molecular target. These examples provide support for the validity of discovering modified disease-specific gene products that can serve as drug targets and be used to prescreen patients via diagnostic platforms capable of identifying those eligible for target-specific therapy.

Other personalized platforms include those that monitor gene expression profiles or soluble markers contained within biofluids (serum, plasma, urine, sputum, or whole cells) that may serve as surrogates for predictive response to a therapeutic regimen. While the example of type 1 diabetes fits into this category, broader discovery approaches are being pursued in oncology. These include biomarker signature profiles within disease tissue that may predict response to certain chemotherapeutic regimens as well as modifier genes that may predict response to a targeted therapy (7). The use of tumor-specific biomarker signature profiles has been widely pursued in breast cancer based on early successful prognostic and therapeutic paradigms that relied on tumor stage and grade, as well as HER2, estrogen (ER), and progesterone (PR) receptor expression status. Subsequent efforts have further refined breast cancer marker profiling to guide best course of therapy (8). This was achieved by development of a molecular signature panel comprising 21 genes, called Oncotype DX^®^ (9), and subsequently MammaPrint™ (10), which comprises a 70 gene set. In both cases, surrogate gene expression profiles are measured to predict a patient’s prognosis and guidance for use of existing therapies. More recently, a similar product for patient prognosis in colorectal cancer (OncoDX) has been developed (11). While these molecular signatures have value in predicting an individual’s prognosis, they cannot predict potential clinical responses after specific targeted therapy. Despite their success and widespread use in breast or colorectal cancer for clinical follow-up after initial diagnosis, the generation and validation of these gene product signatures has taken a significant amount of time and effort before achieving clinical utility in managing personalized treatment for cancer patients.

There are currently 19 FDA approved companion diagnostic (CDx) assays, 18 of which are approved in oncology (Table 1). Ten of the 18 CDx assays are various qualitative assays for detecting HER2 expression or amplification in breast cancer; 2 are qualitative assays for BRAF V600E mutations; along with qualitative assays for ALK translocations, KRAS mutations and c-KIT; and 3 qualitative assays for EGFR mutations. The relative paucity of FDA approved CDx assays might reflect the difficulty and complexity in requirements for approval of such assays but might also be a reflection of a lack of in-depth knowledge of the underlying biology of cancer and/or the drug target interaction. Further, it is interesting to note that only 3 of the 18 (17%) oncology CDx assays are quantitative in format. While quantitative assays are not necessarily required, this undoubtedly speaks to the differences in regulatory requirements for quantitative relative to qualitative assays, at least with respect to CDx assays.

**Table 1:** **FDA approved companion diagnostics (CDx)**.

Drug trade name (generic name)	Device trade name	Device manufacturer	Approved	Technology/indication
Erbitux (cetuximab)	therascreen KRAS RGQ PCR Kit	Qiagen	2012	Qualitative RT-PCR/CRC

Erbitux (cetuximab); Vectibix (panitumumab)	DAKO EGFR PharmDx Kit	Dako	2006	Qualitative IHC/CRC

Exjade (deferasirox)	Ferriscan	Resonance Health Analysis Services	2013	FerriScan R2-MRI/thalassemia

Gilotrif (afatinib)	Therascreen EGFR RGQ PCR Kit	Qiagen	2013	Qualitative RT-PCR/NSCLC

Gleevec/glivec (imatinib mesylate)	Dako C-KIT PharmDx	Dako	2012	Qualitative IHC/GIST

	INFORM HER2/neu	Ventana Medical Systems	2000	Qualitative FISH/breast cancer
	PathVysion HER2 DNA Probe Kit	Abbott Molecular	2013	Qualitative FISH/breast cancer
	PATHWAY anti-HER2/neu	Ventana Medical Systems	2013	Semi-quantitative IHC/breast cancer
	InSite HER2/neu Kit	BioGenex Laboratories	2005	Semi-quantitative IHC/breast cancer
Herceptin (trastuzumab)	SPOT-Light HER2 CISH Kit	Life Technologies	2012	Quantitative CISH/breast cancer
	Bond Oracle Her2 IHC System	Leica Biosystems	2012	Semi-quantitative IHC/breast cancer
	HER2 CISH PharmDx Kit	Dako	2013	Quantitative ISH/breast cancer
	INFORM HER2 Dual ISH DNA Probe Cocktail	Ventana Medical Systems	2013	Two-color ISH/breast cancer

Herceptin (trastuzumab) Perjeta (pertuzumab)	HercepTest	Dako	2013	Semi-quantitative IHC/breast cancer, metastatic gastric, or gastroesophageal junction adenocarcinoma

KADCYLA (ado-trastuzumab emtansine)	HER2 FISH PharmDx Kit	Dako	2013	Quantitative FISH/breast cancer, metastatic gastric, or gastroesophageal junction adenocarcinoma

Mekinist (tramatenib); Tafinlar (dabrafenib)	THxID™ BRAF Kit	bioMérieux	2013	Qualitative RT-PCR/melanoma

Tarceva (erlotinib)	Cobas EGFR Mutation Test	Roche Molecular Systems	2013	Qualitative RT-PCR/NSCLC

Xalkori (crizotinib)	Vysis ALK Break Apart FISH Probe Kit	Abbott Molecular	2013	Qualitative FISH/NSCLC

Zelboraf (vemurafenib)	Cobas 4800 BRAF V600 Mutation Test	Roche Molecular Systems	2013	Qualitative RT-PCR/melanoma

*Source: http://www.fda.gov/MedicalDevices/ProductsandMedicalProcedures/InVitroDiagnostics/ucm301431.htm*.

Interestingly, many more molecular diagnostic (Dx) assays are in drug labels (Table 2) – not as companion diagnostics but recommended or even required prior to prescribing therapy or for therapeutic monitoring – and some of these have been cleared by the FDA through the 510(k) process (Table 3). Complex pharmacogenomics signatures such as Oncotype DX^®^ and even BRCA mutation analyses (12) are routinely used in clinical oncology but are performed under the CLIA regulation (Clinical Laboratory Improvement Amendments of 1988) and are classified as Laboratory Developed Tests (LDTs). This class of Dx does not go through the rigor of regulatory submissions such as 510(k) or PMA (Pre-Market Approval; a CDx requires a PMA) nor the post-marketing requirements of such assays. The FDA is currently reviewing and is expected to make recommendations in the near future regarding the oversight of LDTs, which may (significantly) change the present landscape.

**Table 2:** **Molecular diagnostics (Dx) in US drug labels**.

Drug	HUGO symbol	Referenced subgroup	Labeling sections
Ado-trastuzumab emtansine	ERBB2	HER2 protein overexpression or gene amplification positive	Indications and usage, warnings and precautions, adverse reactions, clinical pharmacology, clinical studies

Afatinib	EGFR	EGFR exon 19 deletion or exon 21 substitution (L858R) mutation positive	Indications and usage, dosage and administration, adverse reactions, clinical pharmacology, clinical studies, patient counseling information

Anastrozole	ESR1, PGR	Hormone receptor positive	Indications and usage, clinical pharmacology, clinical studies

Arsenic trioxide	PML/RARα	PML/RARα [t(15;17)] gene expression positive	Boxed warning, clinical pharmacology, indications and usage, warnings

Bosutinib	BCR/ABL1	Philadelphia chromosome [t(9;22)] positive	Indications and usage, adverse reactions, clinical studies

Brentuximab vedotin	TNFRSF8	CD30 positive	Indications and usage, description, clinical pharmacology

Busulfan	Philadelphia chromosome	Ph chromosome negative	Clinical studies

Capecitabine	DPYD	DPD deficient	Contraindications, warnings and precautions, patient information

Cetuximab	EGFR	EGFR protein expression positive	Indications and usage, warnings and precautions, description, clinical pharmacology, clinical studies
	KRAS	KRAS codon 12 and 13 mutation negative	Indications and usage, dosage and administration, warnings and precautions, adverse reactions, clinical pharmacology, clinical studies

Cisplatin	TPMT	TPMT intermediate or poor metabolizers	Clinical pharmacology, warnings, precautions

Crizotinib	ALK	ALK gene rearrangement positive	Indications and usage, dosage and administration, drug interactions, warnings and precautions, adverse reactions, clinical pharmacology, clinical studies

Dabrafenib	BRAF	BRAF V600E mutation positive	Indications and usage, dosage and administration, warnings and precautions, clinical pharmacology, clinical studies, patient counseling information
	G6PD	G6PD deficient	Warnings and precautions, adverse reactions, patient counseling information

Dasatinib	BCR/ABL1	Philadelphia chromosome [t(9;22)] positive; T315I mutation posit	Indications and usage, clinical studies, patient counseling information

Denileukin diftitox	IL2RA	CD25 antigen positive	Indications and usage, warnings and precautions, clinical studies

Erlotinib	EGFR	EGFR protein expression positive EGFR exon 19 deletion or exon 21 substitution (L858R) positive	Clinical pharmacology Indications and usage, dosage and administration, clinical pharmacology, clinical studies

Everolimus	ERBB2	HER2 protein overexpression negative	Indications and usage, boxed warning, adverse reactions, use in specific populations, clinical pharmacology, clinical studies

Everolimus	ESR1	Estrogen receptor positive	Clinical pharmacology, clinical studies

Exemestane	ESR1	Estrogen receptor positive	Indications and usage, dosage and administration, clinical studies, clinical pharmacology

Fluorouracil	DPYD	DPD deficient	Warnings

Fulvestrant	ESR1	Estrogen receptor positive	Indications and usage, clinical pharmacology, clinical studies, patient counseling information

Ibritumomab tiuxetan	MS4A1	CD20 positive	Indications and usage, clinical pharmacology, description

Imatinib	KIT	c-KIT D816V mutation negative	Indications and usage, dosage and administration clinical pharmacology, clinical studies
	BCR/ABL1	Philadelphia chromosome [t(9;22)] positive	
	
	PDGFRβ	PDGFR gene rearrangement positive	
	
	FIP1L1/PDGFRα	FIP1L1/PDGFRα fusion kinase (or CHIC2 deletion) positive	Indications and usage, dosage and administration, clinical studies

Irinotecan	UGT1A1	UGT1A1*28 allele carriers	Dosage and administration, warnings, clinical pharmacology

Lapatinib	ERBB2	HER2 protein overexpression positive	Indications and usage, clinical pharmacology, patient counseling information

Letrozole	ESR1, PGR	Hormone receptor positive	Indications and usage, adverse reactions, clinical studies, clinical pharmacology

Mercaptopurine	TPMT	TPMT intermediate or poor metabolizers	Dosage and administration, contraindications, precautions, adverse reactions, clinical pharmacology

Nilotinib	BCR/ABL1	Philadelphia chromosome [t(9;22)] positive	Indications and usage, patient counseling information

Nilotinib	UGT1A1	UGT1A1*28 allele homozygotes	Warnings and precautions, clinical pharmacology

Ofatumumab	MS4A1	CD20 positive	Indications and usage, clinical pharmacology

Omacetaxine	BCR/ABL1	BCR-ABL T315I	Clinical pharmacology

Panitumumab	EGFR	EGFR protein expression positive	Indications and usage, warnings and precautions, clinical pharmacology, clinical studies
	KRAS	KRAS codon 12 and 13 mutation negative	Indications and usage, clinical pharmacology, clinical studies

Pazopanib	UGT1A1	(TA)7/(TA)7 genotype (UGT1A1*28/*28)	Clinical pharmacology, warnings and precautions

Pertuzumab	ERBB2	HER2 protein overexpression positive	Indications and usage, warnings and precautions, adverse reactions, clinical studies, clinical pharmacology

Ponatinib	BCR/ABL1	Philadelphia chromosome [t(9;22)] positive, BCR–ABL T315I mutation	Indications and usage, warnings and precautions, adverse reactions, use in specific populations, clinical pharmacology, clinical studies

Rasburicase	G6PD	G6PD deficient	Boxed warning, contraindications

Rituximab	MS4A1	CD20 positive	Indication and usage, clinical pharmacology, description, precautions

Tamoxifen	ESR1, PGR	Hormone receptor positive	Indications and usage, precautions, medication guide
	F5	Factor V Leiden carriers	Warnings
	F2	Prothrombin mutation G20210A	

Thioguanine	TPMT	TPMT poor metabolizer	Dosage and administration, precautions, warnings

Tositumomab	MS4A1	CD20 antigen positive	Indications and usage, clinical pharmacology

Trametinib	BRAF	BRAF V600E/K mutation positive	Indications and usage, dosage and administration, adverse reactions, clinical pharmacology, clinical studies, patient counseling information

Trastuzumab	ERBB2	HER2 protein overexpression positive	Indications and usage, warnings and precautions, clinical pharmacology, clinical studies

Tretinoin	PML/RARA	PML/RARα [t(15;17)] gene expression positive	Clinical studies, indications and usage, warnings

Vemurafenib	BRAF	BRAF V600E mutation positive	Indications and usage, warning and precautions, clinical pharmacology, clinical studies, patient counseling information

Source: http://www.fda.gov/Drugs/ScienceResearch/ResearchAreas/Pharmacogenetics/ucm083378.htm

**Table 3:** **FDA 510(k) cleared molecular diagnostics (Dx)**.

Disease state	Device	Year	Device manufacturer	Comments
AML	Vysis EGR1 FISH Probe Kit	2011	Abbott Molecular	Deletions in EGR1; bone marrow specimens; aid in prognosis

B-cell CLL	Vysis CLL FISH Probe Kit	2011	Vysis	Deletions in TP53, ATM, and D1I3S319 and gain in D1I2Z3; peripheral blood; aid in prognosis
	CEP 12 DNA Probe	1997	Vysis	FISH; specific for centromere 12; peripheral blood; prognosis

Bladder cancer	Vysis UroVysion Bladder Cancer Recurrence Kit	2004	Vysis	Aneuploidy of chromosomes 3, 7, 17 and loss of 9p21 locus; urine specimens; TCC; monitor recurrence

Breast cancer	MammaPrint	2011	Agendia	Gene expression profile; fresh frozen tissue; assess risk for distant metastasis and prognosis
	GeneSearch Breast Lymph Node (BLN) Test Kit	2009	Veridex	Gene expression panel; metastasis in lymph nodes; aids in the decision to excise additional lymph nodes and staging
	Dako TOP2A FISH PharmDx Kit	2012	Dako	FISH to detect copy number changes of TOP2A; FFPE; prognosis in high risk breast cancer patients

Cystic fibrosis	eSensor CF Genotyping Test	2009	Osmetech Molecular Diagnostics	Detects a panel of mutations and variants in CFTR; genomic DNA; genetic carrier screening
	xTAG Cystic Fibrosis 60 Kit v2	2009	Luminex Molecular Diagnostics	Detects and identifies a panel of mutations and variants the CFTR; genetic carrier and newborn screening

Prostate cancer	NADiA ProsVue	2011	Iris Molecular Diagnostics	Determines rate of change of total PSA; serum; an aid in identifying those patients at reduced risk for recurrence of prostate cancer
	PROGENSA PCA3 Assay	2012	Gen-Probe	PCA3 and PSA RNA ratio; urine; aids physicians in determining the need for repeat prostate biopsies in men who have had a previous negative biopsy

Tissue of origin	Pathwork Tissue of Origin Test Kit – FFPE	2012	Pathwork Diagnostics	Compares RNA expression patterns in a patient’s FFPE tumor with those in a database; tissue; aid in determining origin of cancer
	Pathwork Tissue of Origin Test	2008	Pathwork Diagnostics	Compares RNA expression patterns in a patient’s fresh/frozen tumor with those in a database; tissue; aid in determining origin of cancer

Source: http://www.fda.gov/MedicalDevices/ProductsandMedicalProcedures/InVitroDiagnostics/ucm330711.htm

## Antibody-Drug Conjugates and Radioimmunotherapies

Recent clinical success of monoclonal antibody (mAb) drug conjugates (ADCs) has spurred the field of highly toxic chemotherapeutic drugs for targeted therapy. The development of ADCs offers dual benefits: the ability to preselect patients whose disease expresses the target antigen for tumor-specific delivery and the opportunity to deliver highly toxic (novel or repurposed) compounds to antigen positive tumors while avoiding toxic off-target effects commonly found with non-targeted SCEs or radionuclides (hereon referred to as cytotoxins) (13). Enhanced technologies that enable robust linkage of a targeting agent to a cytotoxin such as radionuclides, chemotherapeutic SCEs, and gene silencing nucleic acids has led to the establishment of a wide array of next generation targeted therapies (14). The targeting moieties themselves have varied from full-length antibodies to recombinant proteins, small polypeptides, and nanoparticles (NPs). A diversity of linkage chemistries that allow conjugation of a cytotoxin to the targeting agent have been implemented depending upon where in the tissue it is most desirable to have the cytotoxin delivered and, if required, liberated from the targeting agent. With the early success of antibody–cytotoxin conjugates using radionuclides (referred to as radioimmunotherapy, RIT), such as yttrium-90 (^90^Y)-labeled-ibritumomab tiuxetan (15) and iodine-131 (^131^I)-labeled tositumomab (16) in treating refractory lymphoma, as well as the recently approved ADC trastuzumab-DM1 (T-DM1, Kadcyla^®^) (17), and brentuximab vedotin (SGN35, Adcetris^®^) (18), significant progress in personalized medicine has been attained (19). Part of this advancement is due to the improved therapeutic activity over the parental agents (the cytotoxic or targeting agent alone) resulting in a better clinical outcome while minimizing toxicity. Rituximab is a chimeric mouse–human IgG1 mAb directed to the CD20 cell surface protein and approved for treatment of B-cell lymphomas. ^90^Y-labeled-ibritumomab tiuxetan and I^131^-labeled tositumomab, both of which also target CD20, showed statistically significant clinical responses in patients as compared to rituximab or chemotherapy alone and were approved for use in rituximab-refractory patients. Unfortunately, the application of these RITs in clinical practice has been limited by the complexity of handling β-emitting radionuclide-labeled mAbs before and after patient treatment. These limitations have fostered the generation of alternate molecules, including alpha emitting RITs (20) as well as non-radioactive cytotoxins that can be more practically conjugated to mAb, protein, or peptide-based targeting agents, without affecting their pharmacokinetic or pharmacodynamic properties (i.e., diminished ability to maximally and specifically access its target expressed by the diseased tissue). While therapeutic improvements have been reported in cancers using RITs and ADCs vs. non-conjugated agents, not all patients treated with RIT or ADC agents have shown enhanced benefit, suggesting diagnostic opportunities for improving the therapeutic use of conjugates (21). Table 4 provides an overview of approved ADCs and RITs.

**Table 4:** **Examples of approved antibody-drug conjugates (ADCs) and radioimmunotherapeutics (RIT’s) in oncology**.

Trade name (generic)	Manufacturer	Target	Conjugate	Approved	Indication	Comments
**ANTIBODY-DRUG CONJUGATES (ADCs)**						
**Mylotarg**
Gemtuzumab ozogamicin	Pfizer/Wyeth	CD33	Calecheamicin	2001	Recurrent AML (age 60+)	Voluntarily withdrawn in 2010, due to product safety issues and lack of clinical benefit

**Adcetris**
Brentuximab vedotin	Seattle Genetics	CD30	Mono-methyl auristatin E (MMAE)	2011	Refractory Hodgkin’s lymphoma	
					Refractory systemic anaplastic large cell lymphoma

**Kadcyla**
Trastuzumab emtansine	Genentech/Roche	Her2/neu	Maytansinoid DM1	2013	HER2-positive metastatic breast cancer	Approved for patients who have received prior treatment with Herceptin^®^ (trastuzumab) and a taxane chemotherapy

Trade name (generic)	Manufacturer	Target	Isotope	Approved	Indication	Comments

**RADIOIMMUNOTHERAPEUTICS (RITs)**						
**Zevalin** Ibritumomab tiuxetan	Biogen-Idec/Spectrum pharmaceuticals	CD20	^90^Y	2002	Recurrent, low-grade or follicular B-cell non-Hodgkin’s lymphoma

**Bexxar**
Iodine (131I) tositumomab	Corixa/GSK	CD20	^131^I	2003	CD20 positive, follicular NHL, refractory to rituximab and relapsed following chemotherapy	Manufacture discontinued in 2014 due to poor sales

As indicated above, continued improvement and development of targeted therapies, using ADCs, RITs, or other technologies, is required but not sufficient to realize the maximal therapeutic potential of personalized medicine. Tailoring of a therapy to an individual’s cancer requires knowledge of the underlying biology of that cancer and may involve utilizing surrogate molecular signatures, drug target expression profiles, and/or degree of targeted conjugate uptake for predicting patient response. The heterogeneity described for individual tumors (22) only adds to the complexity of defining the biomolecular characteristics of a patient’s malignant tissue and selecting a therapy, or combination of therapies, most likely to be effective for the individual patient. In turn, such tumor heterogeneity adds to the complexity of development of the requisite surrogate Dx or CDx that is required to maximize the benefit of targeted therapies. It is generally accepted that the more complex the diagnostic platform, the more intricate is the regulatory path for approval, especially if the diagnostic is a CDx. A CDx is considered high risk by most regulatory authorities as it specifically dictates therapeutic intervention, in contrast to Oncotype Dx, MammaPrint, or OncoDX type diagnostic tools that merely supply information relative to prognosis and guide the therapeutic intervention. The development, analytical, and clinical validation of complex multi-marker diagnostic biomarker signatures is both time consuming and expensive. In addition, the alignment of therapeutic-diagnostic development timelines is challenging at best, especially if such signatures are not discovered until *post hoc* analysis of Phase 2 clinical trials. The recent clearance by the US FDA of next generation sequencing (NGS) instrumentation for cystic fibrosis is an important step for the use of new technologies to support complex assay developments, particularly as they relate to oncology where such complex signatures are likely required (23). However, as noted, biomarker signatures for predicting response to a given therapy may not simply involve gene expression or mutation profiles but, rather, complex gene product expression profiles.

## Targeted Cytotoxic Agents – TCAs

Despite the successful demonstration that targeted cytotoxic agents (TCAs), such as ADCs and RITs, can provide added clinical benefit for certain cancers, a number of challenges still remain for their clinical success across a broad spectrum of cancer indications. The effectiveness of targeted cytotoxin conjugates depends in part on the inherent features of the conjugate used. Some of the TCA properties that can be optimized include: (1) tumor recognition and penetration; (2) serum half-life to minimize liberation of the cytotoxin in serum that may result in off-target effects; (3) targeting epitopes on a cell surface antigen that can support maximal conjugate internalization; (4) ability of the targeting agent to retain its target specificity in the conjugated form; and (5) large-scale conjugation of the cytotoxin to the targeting moiety for GMP manufacturing at a reasonable cost-of-goods. Smaller molecular weight targeting conjugates that employ antibody fragment or peptide platforms offer the opportunity to improve TCA tumor penetration (21), enhance binding specificities (24) and internalization (25), as well as lower serum half-lives to avoid prolonged circulation (26). Furthermore, smaller sized TCAs offer the ability to employ alternative manufacturing approaches to minimize cost-of-goods in contrast to mammalian cell fermentation that is required for manufacturing of full-length monoclonal antibodies. While antibody and antibody fragment conjugates appear to offer additional benefits for developing disease-specific therapies, the limited frequency in which a cell surface target is strictly expressed across heterogeneous disease vs. normal tissue remains a major drawback. In cancer, several cell surface targets have been identified that appear to be tumor-specific but the frequency of expression is quite variable from one tumor type to another thereby limiting the breadth by which an approved TCA can be used across different cancer indications (27). Furthermore, recent studies have demonstrated that the expression levels and/or distribution of cell surface targets on tumor cells or tumor-associated stromal cells can vary within the same specimen (28). Hence, the development of high affinity and high specificity targeting agents to maximize tumor recognition in cases of low and heterogeneous target expression is needed. The number of broadly expressed molecular targets that are present on a diseased cell and not on normal tissues that can be selectively targeted by a TCA is low. Nevertheless, several disease-specific antigens have been identified as a result of epigenetic mechanisms, alternative splicing, gene rearrangement, and overexpression that support the potential use of this class for maximizing the therapeutic potential of targeted agents (27). As efforts continue across the industry to identify more disease-specific targets via a variety of genomic and proteomic discovery approaches (discussed below), more broadly expressed disease-associated targets and disease targeting agents have been identified from the screening of naturally occurring pathogenic proteins, intra-protein domains, and NPs scaffolds (29–31). These platforms offer the potential to employ theranostics: the co-development of a TCA along with the same targeting vector linked to a diagnostic agent to determine effective targeting and patient selection (32). Moreover, the use of TCA formats enables the potential repurposing of pharmacologically defined cytotoxic agents on the market, which may lead to faster development timelines of TCA by leveraging prior clinical experience, or the salvaging of compounds that showed anti-tumor activity in clinical trials but failed due to off-target toxicities. One should also bear in mind that, while the expression of the target is required, it may not be sufficient for long-lasting responses. In fact, due to the inherent heterogeneity of tumors and potential escape mechanisms [as seen for example with BRAF inhibitors (33)], theranostics and the TCA strategy in general would likely benefit from being combined with other drugs that have different mode of action and/or target.

## Nanoparticles and Aptamers

Over the past two decades, the use of NPs has shown promise in delivering therapeutic drugs to malignant cells. Early NP-derived agents were primarily designed by optimizing particle size, chemical composition (lipids, silica, nucleic acids), and charge in an attempt to deliver tumor-specificity (34). Next generation NP technologies incorporated the use of disease-specific ligands, such as antibodies and aptamers, which could bind to disease-associated cell surface receptors and deliver therapeutic SCEs. Unfortunately, as mentioned above, the discovery of widely expressed disease-specific receptors that can mediate robust internalization are infrequent. More recently, aptamer-bound NPs have been found to be useful in delivering cytotoxic agents to cancers by targeting disease-specific epitopes on cell surface tumor antigens (35). Peptide aptamers are combinatorial protein molecules usually consisting of short peptides inserted within a scaffold protein resulting in conformational assortment that creates a target-binding diversity. Nucleic acid-based aptamers can achieve similar levels of conformational diversity and target specificity as peptide-based aptamers. Since nucleic acids are also being explored as NP to carry, deliver, and release chemotherapeutic agents, they may represent unique building blocks for both aptamers and NPs manufacturing. The use of aptamers expands the ability to identify subtle differences in the topographical structure of cell surface tumor antigen motifs that are not as easily recognized by traditional proteomic platforms. In light of their size, aptamer-guided NPs have been further engineered as theranostics, whereby the NP contains the targeting aptamer, a cytotoxic agent and an imaging agent that can monitor tumor uptake directly in the patient (36). Patients showing tumor-specific uptake are then deemed suitable for NP-cytotoxic therapy while those that do not can pursue other therapeutic options. Again, despite these promising results, challenges still remain in aptamer-guided NP theranostics including non-specific NP tissue binding, systemic stability, GMP manufacturing, and broad-based application to multiple cancer types. It is worth noting that the successful development of targeted NPs will be demonstrated through a combination of target specificity, a high tumor-to-normal tissue ratio and affinity that will enable the agent to “find and bind” low target expression to deliver their cytotoxic payload. Similarly, these properties are required for their use in diagnostic modalities including patient selection and monitoring of therapeutic efficacy. Table 5 contains a list of marketed and clinical stage NPs being developed for oncology.

**Table 5:** **Examples of clinical stage nanoparticles and naturally occurring proteins in development for oncology**.

Organization	Compound name	Compound description	Target/active agent	Development stage
Alnylam Pharmaceuticals	ALN-VSP	Liposomal based nanoparticle containing siRNA	KSD and VEGF/siRNA	Phase 1

BIND Biosciences	Bind-014	Polylactide–polyethylene glycol biopolymer nanoparticle containing a chemotoxin and targeting ligand	PSMA/docetaxel	Phase 2

Celgene	Nab-paclitaxel	Albumin based nanoparticle	Paclitaxel	Approved (Abraxane^®^)

Cerulean Pharma	CRLX-101	Cyclodextrin-based nanoparticle encapsulating a chemotoxin	Camptothecin	Phase 1/2

Janssen Pharmaceuticals	Pegylated liposomal doxorubicin	Pegylated liposomal nanoparticle containing a chemotoxin	Doxorubicin	Approved (Doxil^®^)

Morphotek	TM601	36 Amino acid peptide from scorpion venom that binds transformed cells and tumor endothelial cells via annexin A2 complex	Annexin A2 complex	Phase 1 (naked peptide format)

University of Illinois at Chicago	NSC745104	28 Amino acid fragment of the protein cupredoxin azurin from pseudomonas aeruginosa that increases intracellular p53 concentrations	p53	Phase 1

*KSD, kinesin spindle protein; PSMA, prostate-specific membrane antigen; VEGF, vascular endothelial growth factor*.

## Natural Agents Targeting Tumors

Several natural agents are able to target differentially expressed or conformation-specific cell surface antigens that are not easily identified by nucleic acid or proteomic analyses nor are easily targeted using traditional protein/antibody approaches. In particular, proteins, toxins or metabolites contained within plants, insects, arthropods, reptiles, viruses, and bacteria have yielded a number of agents capable of binding to specific host cell surface and intracellular proteins as a means to defend against predators and/or suppress their immune system as well as paralyze or even kill their prey (37, 38). Biochemical studies using natural agents from these sources have found them to have disparate activities. These include those that bind and are retained on the cell surface to suppress the activity of enzymes and channels while others have been shown to internalize upon binding to cell type-specific cell surface proteins to suppress intracellular functions. Naturally occurring polypeptides (NOP) from these sources include the following agents: vacuolating toxin A (VacA), which enters human cells via sphingomyelin (39); hepatitis C viral coat protein, which enters cells via claudin-1 (40); *Clostridium perfringens* enterotoxin, which binds to claudin-4 and causes cytotoxicity in cancer cells (41); crotamine, a toxin from rattlesnake venom that enters cells via heparin sulfate proteoglycans (42); and cholorotoxin, which binds to activated epithelial cells and internalizes via the annexin A2 complex (43, 44). Upon further experimental validation of tumor selectivity, any of these agents may serve as potential targeting moieties in the context of a TCA and could also be incorporated into theranostics platforms.

A critical factor for a therapeutic conjugate to provide clinical benefit is the ability to be systemically maintained at a certain molar level in order for the drug to effectively reach the target cells and accumulate at a concentration sufficient for the cytotoxin to exert its pharmacologic effect. Potential drawbacks of using NPs are their relative short serum and intracellular half-lives making extensive dosing and formulation studies critical for their success (45). Alternatively, NOPs have been selected by nature for their ability to impact cellular targets and maintain their function upon exposure. Moreover, their structures have evolved to withstand systemic degradation and immune responses by the host’s serum proteolytic and host defense systems. These features along with their small molecular size warrant exploring NOP potential applications in theranostics platforms and large-scale manufacturing for development. Furthermore, NOPs could find applications in immunohistochemistry and potentially in biofluid-based assays for soluble ligands or in the detection of specific circulating tumor cells (CTCs).

Screening of natural peptides and cell penetrating peptides may offer an advantage for rapid development of targeted therapy by using cell-specific uptake as a predictive marker for therapeutic benefit. This approach may offer a more rapid approach for developing personalized medicines over traditional molecular association, pharmacogenomic, and/or gene signature platforms that may require longer timelines for discovery and validation. However, the use of the latter can be combined during development once biomarker signature profiles have been reproducibly validated for predictive responses to treatment for a disease or disease-specific targeting agent. Table 5 contains a list of some NOPs being developed for oncology.

## Example of Naturally Occurring Polypeptide: Chlorotoxin

Chlorotoxin (CTX) is one of several NOPs that may be utilized for development of theranostics. Protein fractions of the venom from the Israeli scorpion, *Leirius quinquestriatus* were screened for the ability to selectively suppress tumor cell growth while leaving normal epithelial cells unaffected (46). This effort identified a 36 amino acid peptide (CTX) that is able to preferentially bind tumor vs. normal epithelial cells and perturb cell growth. Molecular studies have suggested that CTX binds and internalizes into a wide range of tumor types via the annexin A2 complex, a ubiquitously expressed intracellular protein in normal cells that is found expressed in complexes on the exterior surface of extracellular membranes of transformed cells (44). Preclinical studies have shown the ability of CTX to deliver radionuclides, complex dyes, as well as NPs preferentially to tumor cells *in vitro* and *in vivo* (47). In addition, preclinical studies have also shown CTX to have antiangiogenic activity as a naked peptide (48). Synthetic CTX has been tested in clinical trials and has shown the ability to deliver specifically conjugated ^131^I-radionuclide to tumors after local or systemic delivery with no detectable uptake observed in normal tissues (49) as well as affect angiogenesis as a naked peptide in a Phase 1 trial. The platform is currently being expanded for diagnostic applications (49) and for delivery of cytotoxic compounds including radionuclides to tumors (unpublished observations). The preclinical and clinical studies reported to date suggest a natural compound like CTX could be formatted as a theranostic agent to support personalized medicine. This would be beneficial in light of the broad presence of cell surface annexin A2 complexes in transformed vs. normal cells.

Several studies have provided examples of how CTX could potentially be used as a theranostic agent for cancer. These include imaging studies demonstrating CTX conjugate localization to tumors vs. normal tissues. In one format, CTX has been developed as an imaging bioconjugate composed of CTX and Cy5.5, a fluorescent agent that emits photons in the near-IR spectrum (49). The bioconjugate has been shown to be useful in detecting cancer foci and metastases non-invasively under surgical operating conditions. Systemically, a CTX radionuclide conjugate has been shown to detect metastatic lesions in cancer patients with peripheral and CNS disease (50). Ongoing efforts are aimed at selecting the payload(s) with theranostic features as well as optimizing conjugation chemistry for delivering both a cytotoxin and a tracer to the tumor site.

## Theranostics Challenges and Opportunities: Use of Co-Developing Diagnostic and Therapeutic Targeting Vectors

**Figure 1:**
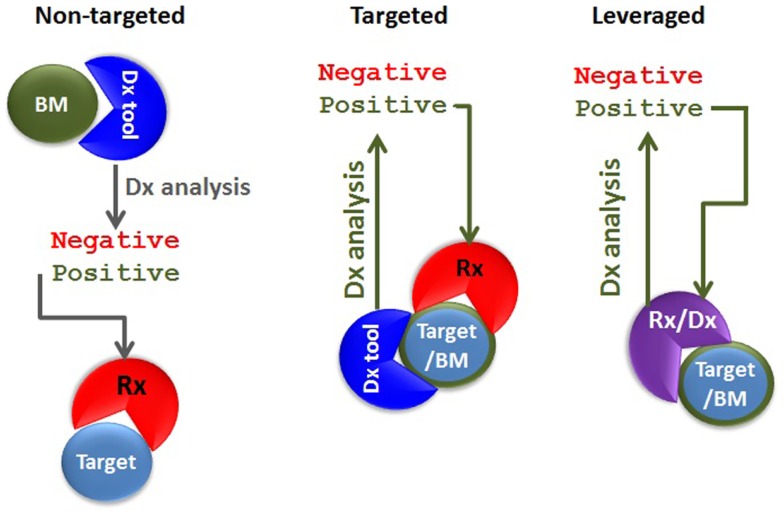
**Types of theranostic agents**. In non-targeted theranostics (left panel), the biomarker (BM), the diagnostics (Dx) tool, the target, and the therapeutic agent (Rx) are separate entities. In the targeted theranostics (middle panel), the target and the biomarker are one and the same, but the Dx tool and the Rx are separate entities. In the leveraged theranostics (right panel), the diagnostic and the therapeutic agent share molecular components.

The definition of theranostics (the merging of the words therapeutics with diagnostics) can be very broad. In its most traditional sense of diagnostics-guided therapy, the concept of theranostics can be identified in the previously cited example, whereby glucose blood levels are measured to determine the timing of the treatment as well as which patients will benefit the most from a hormone (insulin)-based therapy. Nowadays, detecting BRAF mutation is equivalent to measuring blood glucose in that it allows identifying which cancer patients would benefit from an anti-cancer agent targeting a specific kinase (e.g., targeted theranostics against mutated BRAF). In both examples, the diagnostic tool and the therapeutic agent are completely different because they share no molecular components. On the other hand, these two examples differ at least in one regard: while glucose levels represent a “non-targeted” biomarker of the disease state (the true therapeutic target being insulin receptor), detection of BRAF mutation underpins the very presence of the target against which the targeted therapeutic agent has an inhibitory effect. The mutated BRAF constitutes both a therapeutic target as well as a disease biomarker. In another incarnation of targeted theranostics, the targeting agent could be employed in both the diagnostics and therapeutics strategy. In this instance, the diagnostic and the therapeutic agents do share at least one molecular component. Let us call it “leveraged theranostics.” Hence, we can describe at least three different theranostics classes: non-targeted, targeted, and leveraged (Figure 1).

**Figure 2:**
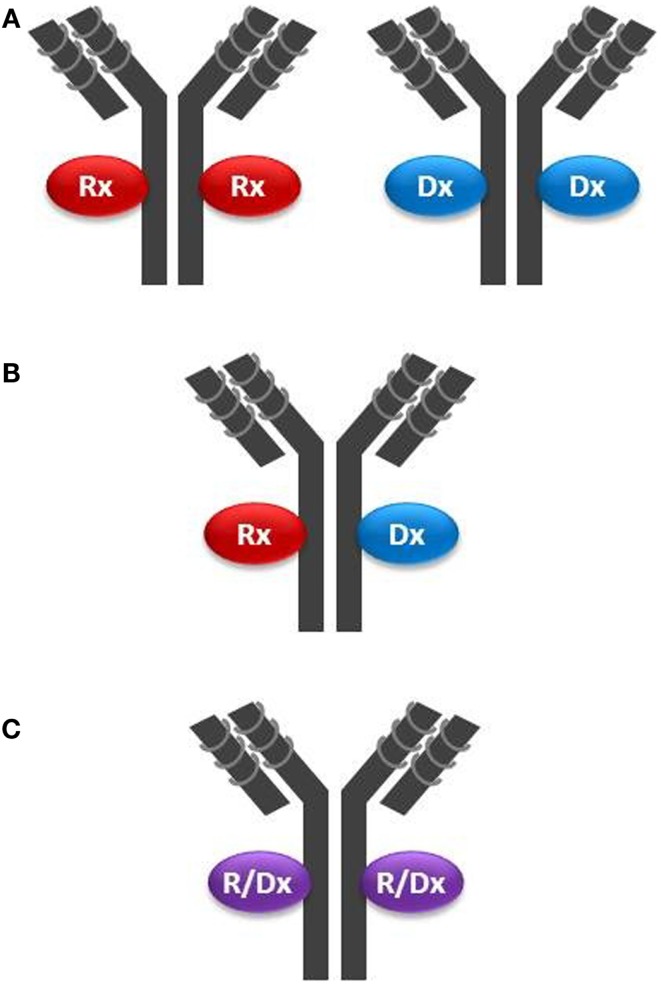
**Configurations of theranostic agents: (A) two separate batches of the same targeting moiety (in this example a mAb) are conjugated with either a therapeutic agent (Rx) such as a cytotoxin, or a diagnostic agent (Dx) such as a radionuclide; (B) the same batch of a mAb is conjugated with *both* Rx and Dx agents on the same targeting molecule; and (C) the same batch of a mAb is conjugated with an agent having both Rx and Dx properties (R/Dx), such as Lutetium-177, on the same targeting molecule**.

Monoclonal antibodies, antibody fragments, NPs, or NOPs may be armed with payloads and deployed in a leveraged theranostic strategy through the clever use of chemical linkers or couplers. One can envision three basic configurations for a leveraged theranostic compound: (1) one payload for therapy and one for diagnostics, with these payloads conjugated on different batches of the same targeting molecule; this configuration involves a single manufacturing process for the targeting moiety, and possibly one process for the linker attachment, but two separate processes for the conjugation of the two payload types; (2) one payload for therapy and one for diagnostics co-conjugated on the same targeting moiety; this implies a single compound and manufacturing process and represents a more ideal scenario; and finally (3) a single payload that can be cytotoxic as well as used for tumor uptake monitoring; this configuration allows for a single compound, manufacturing and conjugation process represents the most ideal scenario. An example of the first configuration (Figure 2A) is offered by the use of specific radionuclides. A radionuclide could be optimal for cytotoxicity but suboptimal for imaging, or vice versa. However, even by using currently available radionuclides, a single targeting agent such as CTX could be “functionalized” using a single chelator (hence a single manufacturing process for the targeting, functionalized moiety), and conjugated to Indium-111 for patient selection and Yttrium-90 for delivering cytotoxicity to the tumor by using two separate conjugation processes. Indium-111, while an excellent imaging agent, is not useful for therapy due to its low tissue penetration characteristic. These properties are reversed in Yttrium-90. Other radionucleotide pairs could be selected to satisfy the desired pharmacological as well as pharmacodynamic properties of the theranostic agent being pursued.

In the second configuration (Figure 2B), one where both payloads are co-attached on the same targeting molecule, one needs to be mindful of stereochemical interferences. For example, multiple payloads can disrupt the tumor cell binding activity of small targeting peptides such as CTX (36 amino acids). Structure–activity relationship analysis would need to be conducted to identify the best chemistry and attachment sites on both the targeting and payload molecules. NPs inherently offer the opportunity to carry multiple payloads to the tumor, including cytotoxins and diagnostic agents, but may not be sufficiently tumor-specific unless coupled with a targeting moiety. Another challenge using multiple payloads could be achieving a defined chemical homogeneity necessary for regulatory approval using a cost-effective manufacturing process.

The third configuration type (Figure 2C) has been achieved over the past several years by employing iodine-131, which, as noted above, suffers from the complexity of its handling. Therefore, this configuration could be improved by the selection of optimized radionuclides, their improved manufacturing processes, storage, and handling procedures, and by more sensitive whole-body radio-imaging devices. A new candidate for this mono-payload theranostic strategy is lutetium-177 (51). This radionuclide is a medium-energy β-emitter with a maximal tissue penetration of 2 mm, hence capable of delivering its cytotoxic energy through several cell layers, while potentially having less off-target toxicity than yttrium-90 (12 mm penetration range). Lutetium-177 also emits low-energy γ-rays allowing both imaging and dosimetry (quantitation of delivered or residual dose). Mono-payload, radiolabeled compounds could be used theranostically, whereby: (i) low, diagnostic (non-therapeutic) doses are used for initial assessment of *in vivo* targeting; (ii) sub-therapeutic doses are administered for dosimetry, allowing precise dose selection, and for monitoring potential toxic effect; and (iii) higher, therapeutic doses are administered to continue to monitor toxicity in conjunction with tumor burden (efficacy, acquired resistance) and tumor uptake (disease modifications, such as loss of target). This strategy is already being implemented when using BEXXAR^®^, an iodine-131-labeled antibody targeting CD20 positive B-lymphoma cells. Using dosimetry, physicians can use a low dose (5 mCi) and directly measure this TCA clearance rate. Patients with high tumor burden, splenomegaly, or bone marrow involvement tend to have faster clearance. Hence, the therapeutic dose (up to 90 mCi) can be prospectively individualized by using an equation (52).

By allowing patient selection and efficacy as well as toxicity monitoring, the potential success of pivotal trials using these theranostic strategies will allow the technological advancement and clinical benefit improvement of personalized medicine. Table 6 contains a list of clinical stage theranostics being developed for oncology.

**Table 6:** **Clinical stage theranostics**.

Organization	Rx compound	Dx compound	Targeting moiety	Target	Configuration type	Development stage
Endocyte	Vinca alkaloid	Technetium-99m	Folate	Folate receptors	Figure 2A	Phase 3

Morphotek	Iodine-131	Iodine-131	Chlorotoxin	Annexin A2	Figure 2C	Phase 2

GlaxoSmithKline	Iodine-131	Iodine-131	Tositumomab (murine IgG2a)	CD20	Figure 2C	Approved (Bexxar^®^)

Institut Jules Bordet	Lutetium-177	Gallium-68	Octreotide (somatostatin analog)	Somatostatin receptor	Figure 2A	Phase 2

Peregrine	Neutralizing mAb	F(ab^′^)2-indium-124	Bavituximab	Phosphatidylserine	Figure 2A^a^	Phase 1^b^/Phase 3^c^

Memorial Sloan-Kettering Cancer Center	Iodine-131	indium-124	8H9 (murine IgG1)	B7-H3	Figure 2A	Phase 1

University Medical Centre Groningen	Neutralizing mAb	Zirconium-89	Trastuzumab	HER2	Figure 2A^a^	Phase 1/2

Institut Jules Bordet/Roche	Maytansine	Zirconium-89	Trastuzumab	HER2	Figure 2A	Phase 2

Areva Med LLC	Lead-212	Lead-212	Trastuzumab	HER2	Figure 2C	Phase 1

^a^Rx compound is a naked chimera IgG with target-neutralizing activity;

^b^Dx compound;

*^c^Rx compound*.

## Conclusion and Future Directions

The use of personalized medicine has many attributes that make the practice invaluable to patients, the pharmaceutical industry, and the healthcare system. The ability to predefine patients with a high likelihood to respond to a given therapy will provide benefit to all parties. For patients, the ability to predict response will improve therapeutic outcome while avoiding unnecessary treatment with ineffective, potentially toxic drugs and thereby lead to a better quality of life, if not a cure. For the pharmaceutical industry, predictive biomarkers (i.e., informative CDx) may improve the probability of success that a drug will provide meaningful clinical results in trials leading to higher approval rates by regulatory authorities and value-creation for the industry and patients alike. For the healthcare system, the ability to avoid futile, potentially toxic therapies will reduce not only drug costs but overall healthcare costs and potentially improve patient health by identifying agents that have a higher probability of success in treating their specific disease. While these attributes are compelling, the ability to implement platforms to support personalized medicine remains challenging. Attempts to identify disease-specific targets or molecular signatures that can provide predictive response outcomes are ongoing across the pharmaceutical industry and academia alike for many indications. While a few successful examples have been achieved, the majority of development programs are handicapped by the paucity of targets associated with disease as well as the time and effort required to validate molecular signatures that can unequivocally and reproducibly predict patient response to non-targeted SCEs.

As the industry refines its technologies and methods to improve upon personalized medicine, a few platforms exist today that may support this initiative in real-time clinical trials. Of particular note is the use of NPs and NOPs that can be conjugated to a therapeutic agent to improve disease-specific uptake of cytotoxic agents and patient response. As discussed above, the use of theranostic strategies employing, for example, a TCA and its co-developed diagnostic vector for *in vivo* prescreening of patients for tumor-specific uptake, offers the opportunity to identify patients with the highest likelihood of benefiting from the TCA therapy. Real-time theranostic imaging strategies may offer an alternative or supplemental approach to the more time consuming pharmacogenomics and/or molecular marker signature analyses for predicting response, although these approaches may yet prove complementary rather than mutually exclusive. Moreover, the application of NP or NOP containing vectors that enable their use for therapy in a broader range and higher frequency of cancers may offer better options than antibody-based therapies whose target is likely restricted to a few indications or across several indications at a low frequency. Other targeting agents in addition to NP and NOPs have also been formatted to support theranostic therapies. Studies in several cancers have found that cell surface proteins such as the folate receptor alpha (FOLR1), a highly expressed protein on ovarian and other epithelial derived cancers can be exploited in a theranostic context (53–55). Strategies to develop conjugates that can be selectively taken up via FOLR1 have been pursued in clinical trials whereby results from these studies have shown that patients whose tumors with uptake of an imaging-folate diagnostic conjugate have enhanced clinical response to a folate–vinblastine therapeutic conjugate compared to patients who do not have folate diagnostic vector uptake (56). Similar approaches to develop conjugate-imaging/conjugate-therapeutic vector pairs have suggested improved patient selection and therapeutic responses. Other examples of diagnostic and therapeutic targeting vector pairs have employed NP technologies to co-develop complexes containing diagnostic agents and an anti-cancer agent, including siRNAs (57). In all cases, the use of TCA and a co-developed targeting diagnostic vector offer alternative methods for delivering personalized therapies to patients in need of new treatments. The key, therefore, for the successful and continued evolution toward personalized medicine is co-development of both the therapeutic and the diagnostic agents as well as diagnostic modalities beginning at the time of target discovery and preclinical studies and continuing through clinical validation and regulatory approvals.

## Conflict of Interest Statement

The authors declare that the research was conducted in the absence of any commercial or financial relationships that could be construed as a potential conflict of interest.
